# Triple Estimation of Fractional Variable Order, Parameters, and State Variables Based on the Unscented Fractional Order Kalman Filter

**DOI:** 10.3390/s21238159

**Published:** 2021-12-06

**Authors:** Dominik Sierociuk, Michal Macias

**Affiliations:** Institute of Control and Industrial Electronics, Warsaw University of Technology, ul. Koszykowa 75, 00-662 Warsaw, Poland; michal.macias@pw.edu.pl

**Keywords:** fractional calculus, fractional Kalman filter, estimation of fractional order systems

## Abstract

In this paper, a method for states, parameters, and fractional order estimation is presented. The proposed method is an extension of the traditional dual estimation method and uses three blocks of filters with appropriate data interconnections. As the main part of the estimation algorithm, the Fractional Unscented Kalman Filter was used. The proposed Triple Estimation algorithm might be treated as a convenient tool for estimation and analysis of a wide range of dynamical systems with fractional constants or variable order nature, especially when knowledge about the identified system is very restricted and both order and system parameters are unknown. In order to show the performance of the proposed algorithm, sets of numerical results are presented.

## 1. Introduction

Fractional calculus is a generalization of traditional differential calculus for cases where orders of differentiation and integration are real or even complex numbers. The theoretical background for this calculus can be found in [1,2,3,4].

Fractional order calculus is widely used for modelling in many areas of applications, especially in diffusion processes. In [5], the results of successful modelling for a heat transfer process in solid material were presented. Moreover, in [6], similar results for heat transfer in heterogeneous materials, described by anomalous diffusion using a fractional order partial differential equation, were shown. Other authors dealt with the idea of the heat diffusion process in non-homogeneous fractal media (e.g., [7,8]) or even the diffusion of information in social networks [9].

Fractional order calculus was also found to be an efficient tool in signal processing [4,10]. Specifically, the Kalman Filter algorithm was generalized for many classes of fractional order systems [11,12,13]. One of the highly promising areas is to use these algorithms for noise estimation with fractional order dynamics (coloured noises) [14,15]. In [16], a fractional Kalman Filter algorithm was used to estimate the bias of MEMS. A survey of algorithms in the area of fractional order sensing and filtering techniques was presented in [17].

In real applications (particularly in modelling of sensor noise), very often, the order of the system is unknown, which makes the identification problem much more complex and therefore complicated. Specifically, when the rest of the system’s parameters are also unknown, it is caused by the highly nonlinear relation for achieving fractional order. For example, in [18], an advanced numerical optimization algorithm was used to obtain the value of the fractional order, and in [11], the dual estimation method was used to estimate the fractional order of the system. To deal with such a complex problem, we propose a new estimation algorithm for the simultaneous estimation of state variables, system parameters, and fractional order. Such an algorithm will be formulated for one type of variable order definition. It is based on a modification (enlargement) of the classical dual estimation algorithm, which contains two estimation actions (one for states variables and another for system parameters). This approach has been extended to fractional order estimation, finally giving the Triple Estimation algorithm.

The estimation of an unknown fractional order leads to variable order differential equations. The variable-order case is much more complicated and less intuitive than the constant order case. There exist at least six different types of variable-order derivative definitions [19,20,21], but only four of them correspond to clearly defined switching schemes, namely input-reductive, input-additive, output-reductive and output-additive ([22,23,24]). Their equivalent switching schemes allow us to better understand the behaviour of orders varying with each definition.

Due to the possibility of a system’s order varying with time, the fractional variable order definition with the most clear interpretation of its nature in the form of a switching scheme will be applied. In [25,26], the authors showed three switching strategies corresponding to appropriate fractional order derivatives. Moreover, analog models based on proposed switching schemes and its experimental validation were presented in [25,26,27]. This paper is organized as follows. Section 2 recalls a Fractional Variable Order State-Space System. In Section 3, the main result from the Triple Estimation algorithm is introduced, and in Section 4, numerical examples of applications of the proposed Triple Estimation algorithm are presented.

## 2. Discrete Variable Fractional Order State-Space System

We can distinguish a few fractional variable-order definitions by considering only the miscellaneous relation between their orders and samples. In this paper, we take into account an extension of the Grünwald–Letnikov definition, where past samples are calculated with binomial coefficients of the current order, and it is formulated as follows:

**Definition** **1.** *The A-type of fractional variable order difference is defined as follows:*

(1) Δ0AΔkαkfk=1hαk∑r=0k(−1)rαkrfk−r.

Based on this definition, let us consider a linear Discrete Fractional Variable Order State-Space (DFVOSS) A-type system [28]:

(2) Δ0AΔk+1Υk+1xk+1=Axk+Buk,xk+1=hΥk+1Δ0AΔk+1Υk+1xk+1

(3) $$\begin{array}{ccc} & - & \sum_{j=1}^{k+1}{\left(-1\right)}^{j}\Upsilon_{j,k+1}x_{k-j+1}\;, \end{array}$$

(4) $$\begin{array}{ccc} y_{k} & = & Cx_{k}\;, \end{array}$$

where

(5) Υj,k=diagα1,kj⋯αN,kj,

(6) $${}^{A}\Delta^{\Upsilon_{k+1}}x_{k+1}=\left[\begin{array}{c} {}^{A}\Delta^{\alpha_{1,k+1}}x_{1,k+1}\\ \vdots \\ {}^{A}\Delta^{\alpha_{N,k+1}}x_{N,k+1} \end{array}\right]$$

(7) $$h^{\Upsilon_{k+1}}=\mathrm{diag}\left[\begin{array}{ccc} h^{\alpha_{1,k+1}} & \cdots & h^{\alpha_{N,k+1}} \end{array}\right]\;$$

and $\alpha_{i,k}\in R$ is the *i*-th fractional variable order of the system; $u_{k}\in R^{d}$ is a system input; $y_{k}\in R^{p}$ is a system output; $A\in R^{N\times N}$, $B\in R^{N\times d}$, and $C\in R^{p\times N}$ are the state system, input, and output matrices, respectively; $x_{k}\in R^{N}$ is a state vector; *N* is a number of state equations; and *h* is a time sampling.

## 3. Triple Estimation Algorithm Based on UFKF Filter

For simplicity, let us take into consideration one state variable constant order model:

(8) Δ0Δk+1αxk+1=fxk+uk+ωk,xk+1=hα0Δk+1αxk+1

(9) $$\begin{array}{ccc} & - & \sum_{j=1}^{k+1}{\left(-1\right)}^{j}\Upsilon_{j,k+1}x_{k-j+1}\;, \end{array}$$

(10) $$\begin{array}{ccc} y_{k} & = & x_{k}+\nu_{k}\;, \end{array}$$

where α is a fractional system order; *f* is a parameter of the system; $\omega_{k}$ and $\nu_{k}$ are system and output noises, respectively; and $Q_{k}$ and $R_{k}$ are covariance matrices of these two noises.

The estimation problem, which is considered in this paper, is used to estimate unknown state variable $x_{k}$, system parameter *f*, and system order α together.

If only the estimation of the state variable and system parameter is required, joint or dual estimation can be used [29,30]. In joint estimation, the state vector is augmented with the desired parameter, with the dynamics of the parameter assumed to be changing (usually assumed to be constant). For our problem, the system of equations is rewritten in the following form:

(11) Δ0Δk+1αxk+1Δ0Δk+11fk+1=fkxk+uk+ωk0,

which, of course, provides a nonlinear system due to the multiplication of two state variables $f_{k}x_{k}$ and implies the use of nonlinear estimation algorithms. Such a method, even for integer order systems, for more complicated problems is not effective. That is why for more complicated problems, the dual estimation algorithm is used [29].

### 3.1. Dual Estimation Scheme

**Figure 1:**
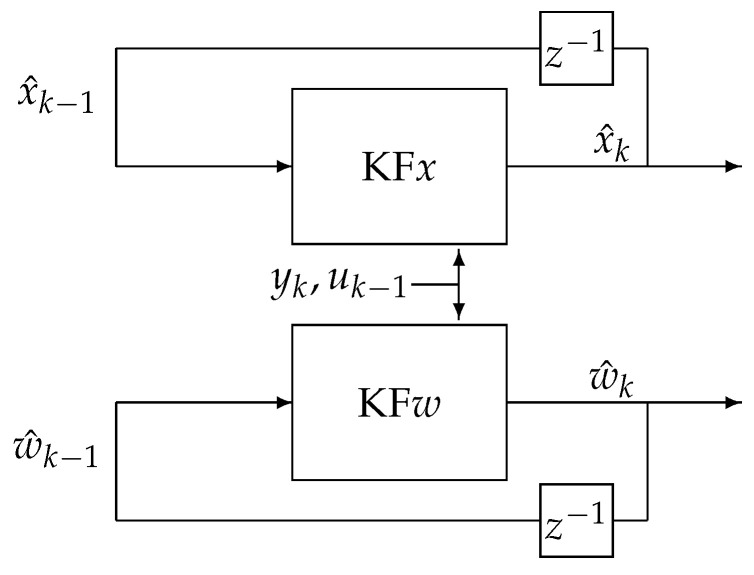
Dual estimation scheme.

The fundamental behaviour of a dual estimation algorithm—separation of parameters and state variables processes—can be reflected in the two main blocks presented in Figure 1. One of them, denoted as KF*x*, is responsible for state variable vector estimation ${\hat{x}}_{k}$, and the second one, denoted as KF*w*, is responsible for estimation of the parameter vector ${\hat{w}}_{k}$. As can be noticed, both the KF*x* and KF*w* filters together estimate the state vector ${\hat{x}}_{k}$ and parameters vectors ${\hat{w}}_{k}$, directly based on the input, the output, and appropriate data interconnections. Therefore, the KF*x* filter estimates the state vector based on the current output $y_{k}$, the past input $u_{k-1}$, the past state vector estimate ${\hat{x}}_{k-1}$, and the past estimated value of parameters vector ${\hat{w}}_{k-1}$ obtained from the KF*w* filter. The next filter, the so-called KF*w*, estimates the parameters vector based on the current output $y_{k}$, the past input $u_{k-1}$, the past parameter vector estimate ${\hat{w}}_{k-1}$, and the past estimated value of state vector ${\hat{x}}_{k-1}$ obtained from the KF*x* filter.

As presented in [11], it is possible to identify a fractional order by dual estimation, but as was recognized during many simulations, it is hard to estimate both the unknown fractional order and system parameters at the same time.

**Figure 2:**
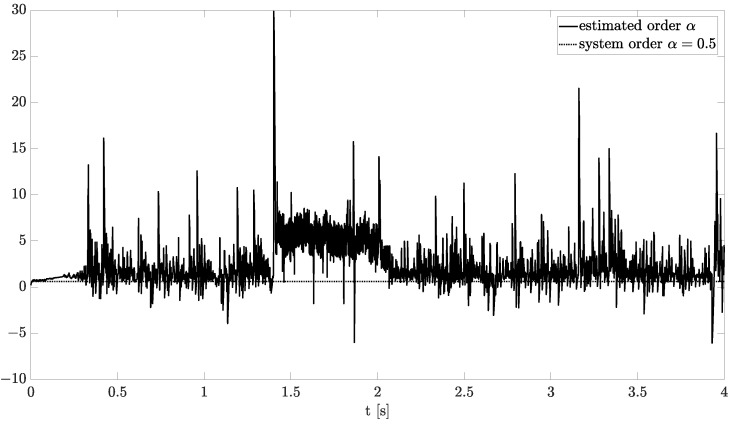
Original and estimated order obtained using the dual estimation algorithm.

**Figure 3:**
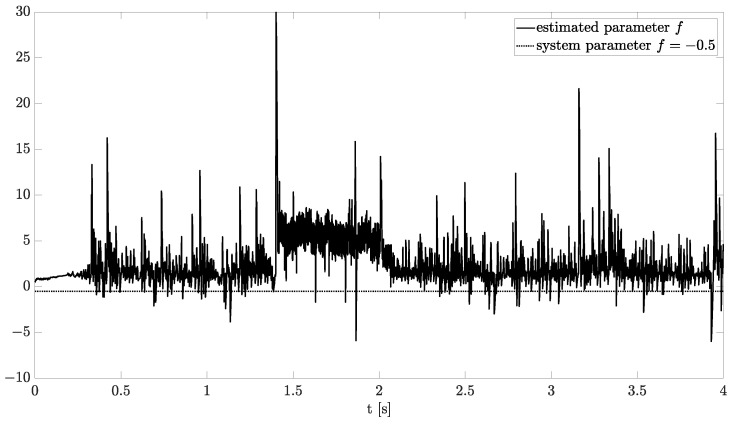
Original and estimated parameter obtained using the dual estimation algorithm.

Exemplary results are presented in Figure 2 and Figure 3. For obtaining these results, the dual estimation algorithm was used when the parameter filter was organized for joint estimation of the order and system parameter. The parameters of the estimated system are given as follows:

(12) $$A=-0.5,B=1,C=1,\alpha_{k}=0.6$$

They are the same as for Example 2, and the same parameters as those of the UKF filter was used. It was tested for different values of parameter δ (0.1,0.5,0.9), and similar, non-acceptable results were obtained.

That is why it could be worth separating the estimation of fractional orders and system parameters in the algorithm. In this paper, the concept of dual estimation is extended in the form of the Triple Estimation algorithm for estimating parameters, the order, and state variables.

### 3.2. Triple Estimation Scheme—The Main Result

The main idea of dual estimation is to separate the state and parameter estimation processes in order to obtain a better control effect for these two processes. However, when the estimation of parameters and order is required, in dual estimation, the estimation action of parameters and order is connected, and there is a problem in obtaining a good enough control effect for these processes (as it was shown in the previous section). In this section, we introduce the Triple Estimation algorithm, which is a generalization of dual estimation algorithm for fractional order estimation presented in [11].

In general, the introduced Triple Estimation algorithm can be treated as an efficient method for states, parameters, and order estimation of fractional order dynamic, simultaneously. Additionally, separation of the order and system parameter estimation processes also allows for better algorithm parameters tuning because we can separately tune the parameters for order and system parameter filters.

**Figure 4:**
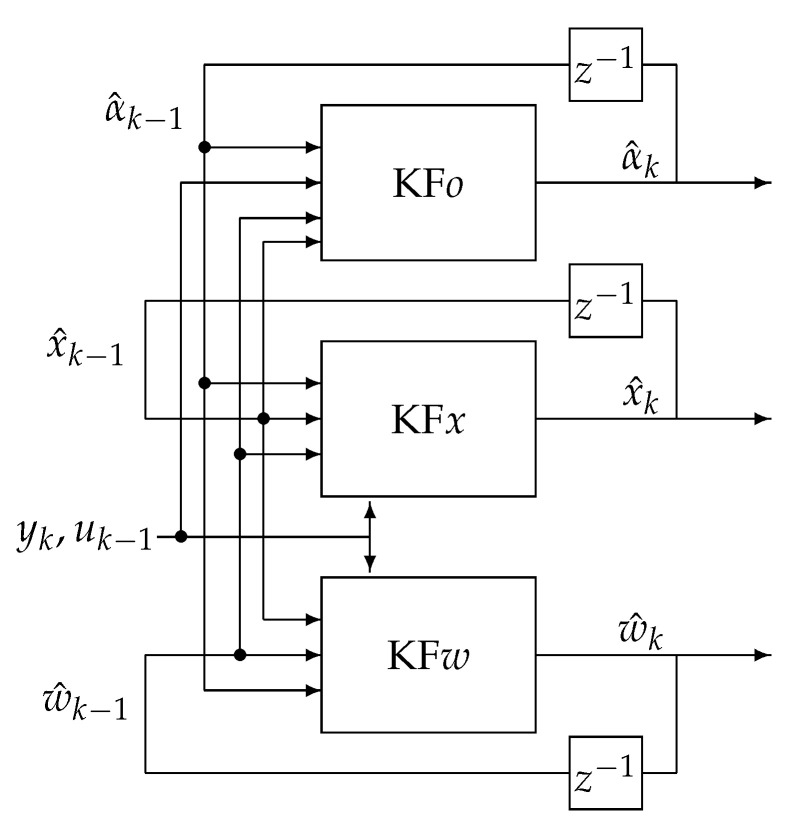
Triple Estimation scheme.

In the Triple Estimation process, the fractional variable order, state variables, and parameter estimation are divided into three estimation actions (filters). The first filter estimates the state variable vector ${\hat{x}}_{k}$, the second one estimates the vector of system parameters ${\hat{w}}_{k}$, and the third filter estimates the fractional variable order. The scheme of this type of estimation is given in Figure 4, where KF*x*, KF*w*, and KF*o* are filters responsible for the state vectors, parameters, and order estimation, respectively.

The filter KF*x* is based on the past estimated value of parameter vector estimates ${\hat{w}}_{k-1}$, data $u_{k-1}$ and $y_{k}$, and past value of estimated order ${\hat{\alpha }}_{k-1}$ obtained from filter KF*o* to evaluate state estimate ${\hat{x}}_{k}$. On the other hand, filter FK*w* uses past estimates obtained by the KF*x* filter, past value of estimated order ${\hat{\alpha }}_{k-1}$ obtained from filter KF*o* and data $u_{k-1}$ and $y_{k}$ to obtain its own state vector and output prediction ${\tilde{\chi }}_{k}^{w}$ and $Y_{k}^{w}$ to extract the next estimate of parameter vector ${\hat{w}}_{k}$. The filter KF*o* is based on estimates from filters KFw and KFx.

#### 3.2.1. Order Estimation Filter KF*o*

Due to the highly nonlinear problem of order estimation, as the KF*o* filter, the Unscented Fractional Variable Order Kalman Filter (similar to the one used in the Dual Estimation algorithm in [11]) was used. The dynamics of order changing was assumed to be constant:

(13) $$\alpha_{k+1}=\alpha_{k}+\omega_{k}^{o},$$

where $\omega_{k}^{o}$ is a noise with variance given by matrix $Q_{k}^{o}$ and represents our knowledge about variability of the order and allow us to set the algorithm for bigger or fewer changes of the estimate during the order estimation sub-process.

The KF*o* algorithm equations are formulated in the following proposition:

**Proposition** **1.** *The Unscented Fractional Variable Order Kalman Filter for the order estimation process (called KFo) in the Triple Estimation algorithm is given by the following set of equations:*

(14) $$\begin{array}{ccc} {\tilde{\alpha }}_{k} & = & {\hat{\alpha }}_{k-1}, \end{array}$$

(15) $$\begin{array}{ccc} {\tilde{P}}_{k}^{o} & = & {\hat{P}}_{k-1}^{o}+Q_{k-1}^{o}, \end{array}$$

(16) $$\begin{array}{ccc} {\tilde{\alpha }}_{k} & = & \left[\begin{array}{cc} {\tilde{\alpha }}_{k} & {\tilde{\alpha }}_{k}\pm {\left(\sqrt{\left(L+\lambda \right){\tilde{P}}_{k}^{o}}\right)}_{i} \end{array}\right], \end{array}$$

(17) $$\begin{array}{ccc} \Delta^{{\tilde{\alpha }}_{k,i}}{\tilde{\chi }}_{k,i}^{o} & = & A\left({\hat{w}}_{k-1}\right){\hat{x}}_{k-1}+Bu_{k-1}, \end{array}$$

(18) χ˜k,io=hα˜k,iΔα˜k,iχ˜k,io−∑j=1k(−1)jα˜k,ijx^k−j,

(19) $$\begin{array}{ccc} {\tilde{Y}}_{k,i}^{o} & = & C{\tilde{\chi }}_{k,i}^{o}, \end{array}$$

(20) $$\begin{array}{ccc} {\tilde{y}}_{k}^{o} & = & \sum_{i=0}^{2L}W^{\left(m\right)}{\tilde{Y}}_{k,i}, \end{array}$$

(21) $$\begin{array}{ccc} P_{y_{k}y_{k}}^{o} & = & \sum_{i=1}^{2L}W_{i}^{\left(c\right)}\left[{\tilde{Y}}_{i,k}-{\tilde{y}}_{k}\right]{\left[{\tilde{Y}}_{i,k}-{\tilde{y}}_{k}\right]}^{T}+R^{o}, \end{array}$$

(22) $$\begin{array}{ccc} P_{\alpha_{k}y_{k}}^{o} & = & \sum_{i=1}^{2L}W_{i}^{\left(c\right)}\left[{\tilde{\alpha }}_{i,k}-{\tilde{\alpha }}_{k}\right]{\left[{\tilde{Y}}_{i,k}-{\tilde{y}}_{k}\right]}^{T}, \end{array}$$

(23) $$\begin{array}{ccc} K_{k}^{o} & = & P_{\alpha_{k}y_{k}}^{o}{\left(P_{y_{k}y_{k}}^{o}\right)}^{-1}, \end{array}$$

(24) $$\begin{array}{ccc} {\hat{\alpha }}_{k} & = & {\tilde{\alpha }}_{k}+K_{k}^{o}\left(y_{k}-{\tilde{y}}_{k}^{o}\right), \end{array}$$

(25) $$\begin{array}{ccc} P_{k}^{o} & = & {\hat{P}}_{k}^{o}-K_{k}^{o}P_{y_{k}y_{k}}^{o}K_{k}^{o}, \end{array}$$

(26) $$\begin{array}{ccc} Q_{k}^{o} & = & \left(1-\delta^{o}\right)Q_{k-1}^{o}+\delta^{o}\left(K_{k}^{o}\right)\left(y_{k}-{\tilde{y}}_{k}^{o}\right){\left(y_{k}-{\tilde{y}}_{k}^{o}\right)}^{T}{\left(K_{k}^{o}\right)}^{T}, \end{array}$$

*where ${\left(\sqrt{\left(L+\lambda \right)P_{k}}\right)}_{i}$ is the i-th column of the matrix square root (e.g., Cholesky factorization), and coefficients of Unscented transformation W are equal to*

(27) $$\begin{array}{ccc} W_{0}^{\left(m\right)} & = & \lambda /(L+\lambda ), \end{array}$$

(28) $$\begin{array}{ccc} W_{0}^{\left(c\right)} & = & \lambda /\left(L+\lambda \right)+\left(1-A^{2}+B\right), \end{array}$$

(29) $$\begin{array}{ccc} W_{i}^{\left(m\right)} & = & W_{i}^{\left(c\right)}=1/\left(2\left(L+\lambda \right)\right), \end{array}$$

*where $\lambda =A^{2}\left(L+\kappa \right)-L$, A is a coefficient describing the width of point expansion during the transformation (in the literature, it is obtained in the range $1\leq A\leq 10^{-4}$, usually denoted as α, but in this article, using an order α, this notation has been changed), κ is an additional scaling coefficient usually chosen as 3-L, B is a coefficient that corresponds with our knowledge about type of noise, and that for Gaussian noise is chosen as B=2 (in the literature, it is usually denoted as β). The δ coefficient is a “forgetting factor” according to the Robbins–Monro stochastic approximation scheme for estimating the innovations (see [31] page 240). The initial values of matrix $P_{0}^{o}$ represents our a priori knowledge about error in choosing the initial value of order $\alpha_{0}$ (we assume that the initial value is different from the original).*

#### 3.2.2. State Estimation Filter KFx

Due to the linear problem of state vector estimation (KFx Filter), the Fractional Variable Order Kalman Filter, given below, is used.

**Proposition** **2.** *The Fractional Variable Order Kalman Filter algorithm for state variable estimation process (called KFx) in the Triple Estimation algorithm is given by the following set of equations:*

(30) Δ0AΔk+1α^kx˜k+1=A(w^k−1)x^k+Buk,

(31) x˜k+1=hα^kΔ0AΔk+1α^kx˜k+1−∑j=1k+1(−1)jα^j,k+1x^k+1−j,

(32) $$\begin{array}{ccc} {\tilde{P}}_{k} & = & \left(h^{{\hat{\alpha }}_{k}}A\left({\hat{w}}_{k-1}\right)+{\hat{\alpha }}_{k}\right)P_{k-1}{\left(h^{{\hat{\alpha }}_{k}}A\left({\hat{w}}_{k-1}\right)+{\hat{\alpha }}_{k}\right)}^{T} \end{array}$$

(33) $$\begin{array}{ccc} & + & Q_{k-1}+\sum_{j=2}^{k}{\hat{\alpha }}_{k-j}P_{k-j}{\hat{\alpha }}_{k-j}^{T}, \end{array}$$

(34) $$\begin{array}{ccc} K_{k} & = & {\tilde{P}}_{k}C^{T}{\left(C{\tilde{P}}_{k}C^{T}+R_{k}\right)}^{-1}, \end{array}$$

(35) $$\begin{array}{ccc} {\hat{x}}_{k} & = & {\tilde{x}}_{k}+K_{k}\left(y_{k}-C{\tilde{x}}_{k}\right), \end{array}$$

(36) $$\begin{array}{ccc} P_{k} & = & \left(I-K_{k}C\right){\tilde{P}}_{k}, \end{array}$$

*where the initial conditions are*

(37) $$x_{0}\in R^{N},\;P_{0}=E\left[\left({\tilde{x}}_{0}-x_{0}\right){\left({\tilde{x}}_{0}-x_{0}\right)}^{T}\right],$$

*and $\nu_{k}$ and $\omega_{k}$ are assumed to be independent with zero expected value.*

#### 3.2.3. Parameters Estimation Filter KF*w*

The problem of parameter estimation is nonlinear, and the Unscented Fractional Variable Order Kalman Filter or Extended Fractional Variable Order Kalman Filter can be used. As we can expect that the Unscented Fractional Variable Order Kalman Filter will give more accurate results, we chose such an algorithm for our filtering process. The dynamics of parameter change was assumed to be constant:

(38) $$w_{k+1}=w_{k}+\omega_{k}^{w},$$

where $\omega_{w}^{o}$ is a noise with variance given by matrix $Q_{k}^{w}$ and represents our knowledge about variability of the parameter. Manipulating matrices $Q_{k}^{w}$ and $Q_{k}^{o}$ allows us to decide if the system parameter or the order will be more sensitive for estimation error. Thus, the filter KF*w* is given as follows:

**Proposition** **3.** *The Unscented Fractional Variable Order Kalman Filter for the parameter estimation process (called KFw) in the Triple Estimation algorithm is given by the following set of equations:*

(39) $$\begin{array}{ccc} {\tilde{w}}_{k} & = & {\hat{w}}_{k-1}, \end{array}$$

(40) $$\begin{array}{ccc} {\tilde{P}}_{k}^{w} & = & {\hat{P}}_{k-1}^{w}+Q_{k-1}^{w}, \end{array}$$

(41) $$\begin{array}{ccc} {\tilde{W}}_{k} & = & \left[\begin{array}{cc} {\tilde{w}}_{k} & {\tilde{w}}_{k}\pm {\left(\sqrt{\left(L+\lambda \right){\tilde{P}}_{k}^{w}}\right)}_{i} \end{array}\right], \end{array}$$

(42) $$\begin{array}{ccc} \Delta^{{\hat{\alpha }}_{k-1}}{\tilde{\chi }}_{k,i}^{w} & = & A\left({\tilde{W}}_{k,i}\right){\hat{x}}_{k-1}+Bu_{k-1}, \end{array}$$

(43) χ˜k,iw=hα^k−1Δα^k−1χ˜k,iw−∑j=1k(−1)jα^k−1jx^k−j,

(44) $$\begin{array}{ccc} {\tilde{Y}}_{k,i}^{w} & = & C{\tilde{\chi }}_{k,i}^{w}, \end{array}$$

(45) $$\begin{array}{ccc} {\tilde{y}}_{k}^{w} & = & \sum_{i=0}^{2L}W^{\left(m\right)}{\tilde{Y}}_{k,i}, \end{array}$$

(46) $$\begin{array}{ccc} P_{y_{k}y_{k}}^{w} & = & \sum_{i=1}^{2L}W_{i}^{\left(c\right)}\left[{\tilde{Y}}_{i,k}-{\tilde{y}}_{k}\right]{\left[{\tilde{Y}}_{i,k}-{\tilde{y}}_{k}\right]}^{T}+R^{w}, \end{array}$$

(47) $$\begin{array}{ccc} P_{w_{k}y_{k}}^{w} & = & \sum_{i=1}^{2L}W_{i}^{\left(c\right)}\left[{\tilde{W}}_{i,k}-{\tilde{w}}_{k}\right]{\left[{\tilde{Y}}_{i,k}-{\tilde{y}}_{k}\right]}^{T}, \end{array}$$

(48) $$\begin{array}{ccc} K_{k}^{w} & = & P_{w_{k}y_{k}}^{w}{\left(P_{y_{k}y_{k}}^{w}\right)}^{-1}, \end{array}$$

(49) $$\begin{array}{ccc} {\hat{\alpha }}_{k} & = & {\tilde{\alpha }}_{k}+K_{k}^{w}\left(y_{k}-{\tilde{y}}_{k}^{w}\right), \end{array}$$

(50) $$\begin{array}{ccc} P_{k}^{w} & = & {\hat{P}}_{k}^{w}-K_{k}^{w}P_{y_{k}y_{k}}^{w}K_{k}^{w}, \end{array}$$

(51) $$\begin{array}{ccc} Q_{k}^{w} & = & \left(1-\delta^{w}\right)Q_{k-1}^{w}+\delta^{w}\left(K_{k}^{w}\right)\left(y_{k}-{\tilde{y}}_{k}^{w}\right){\left(y_{k}-{\tilde{y}}_{k}^{w}\right)}^{T}{\left(K_{k}^{w}\right)}^{T}, \end{array}$$

*where the parameters of an unscented transformation are defined in the same way as in the KFo filter.*

## 4. Numerical Results

This section contains sets of numerical examples applying the Triple Estimation algorithm. The configuration parameters dedicated to state, parameters, and order estimation blocks, of course, have a significant impact on the estimation results. Having separate sets of configuration parameters for each block allows us to influence the appropriate filter and to raise the effectiveness and robustness of the proposed method. However, this task is not straightforward due to the uncertainties appearing in real cases and should be adjusted individually for each fractional order system during analysis. The structure of the simulation models and their sampling time used in the examples correspond to analogue models of fractional order systems validated, e.g., in [11,32,33]. Based on these and considering the clarity of the given results, we decided to use a single input and single output fractional order system as a primary pattern for tests.

All tests were conducted in a Matlab/Simulink environment based on the Fractional Variable-Order Toolkit [34], which was used to simulate the fractional order systems. To highlight the behaviour of the proposed estimation algorithm and its possibilities during an analysis of fractional order systems, all numerical examples were conducted under the following, in common, predefined values:

- Noises parameters
  (52) $$E\left[\omega \omega^{T}\right]=2.5\times 10^{-5},$$
  (53) $$E\left[\nu \nu^{T}\right]=10^{-4},$$
- Parameters of the KF*x* filter
  (54) $$P_{0}=\left[\begin{array}{c} 1 \end{array}\right],Q_{0}=\left[\begin{array}{c} 2.5\times 10^{-5} \end{array}\right],$$
  (55) $$x_{0}=\left[0\right],R=\left[10^{-4}\right],$$
- Parameters of the KF*o* filter
  (56) $$P_{0}^{o}=\left[\begin{array}{c} 0.01 \end{array}\right],Q_{0}^{o}=\left[\begin{array}{c} 0.001 \end{array}\right],$$
  (57) $$\alpha_{0}=\left[1\right],R^{o}=\left[1.25\times 10^{-4}\right],A=1,B=2,\delta^{o}=0.5.$$
- Parameters of the KF*w* filter
  (58) $$P_{0}^{w}=\left[\begin{array}{c} 0.01 \end{array}\right],Q_{0}^{w}=\left[\begin{array}{c} 0.001 \end{array}\right],$$
  (59) $$w_{0}=\left[0\right],R^{w}=\left[1.25\times 10^{-4}\right],A=1,B=2,\delta^{w}=0.5.$$

Additionally, the simulation data were collected with sampling time h=0.001 s and input signal u(t) is the square wave with amplitude equals 1 and frequency equals 1 Hz. To validate the Triple Estimation algorithm, the spread between system order α and system parameter *w* was different in each example. The Examples 1–3, given below, contain a description of the simulated fractional order system and correspond to appropriate plots of state, order, and system parameter estimation based on the proposed algorithm.

**Example** **1.** *Let us consider the linear Discrete Fractional Variable Order State-Space (DFVOSS) A-type system given by Equations (2), (3) and (4), where*

(60) $$A=-0.7,B=1,C=1,\alpha_{k}=0.4$$

**Figure 5:**
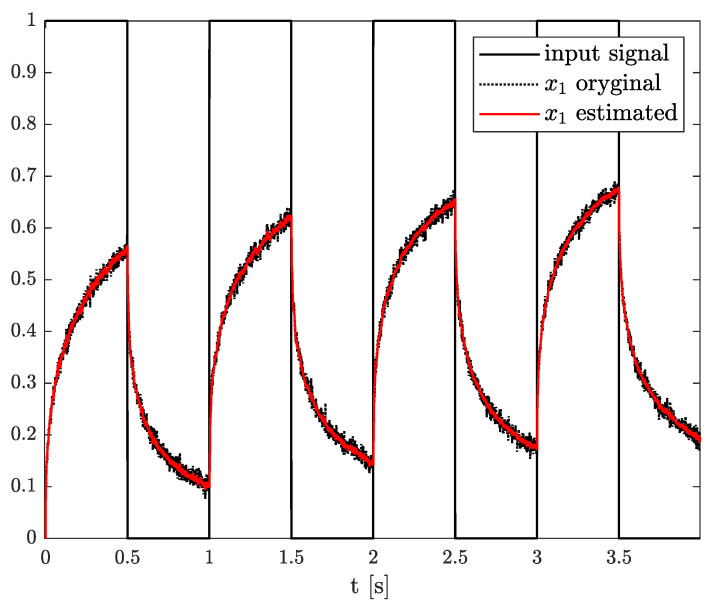
Original and estimated state variables from Example 1.

**Figure 6:**
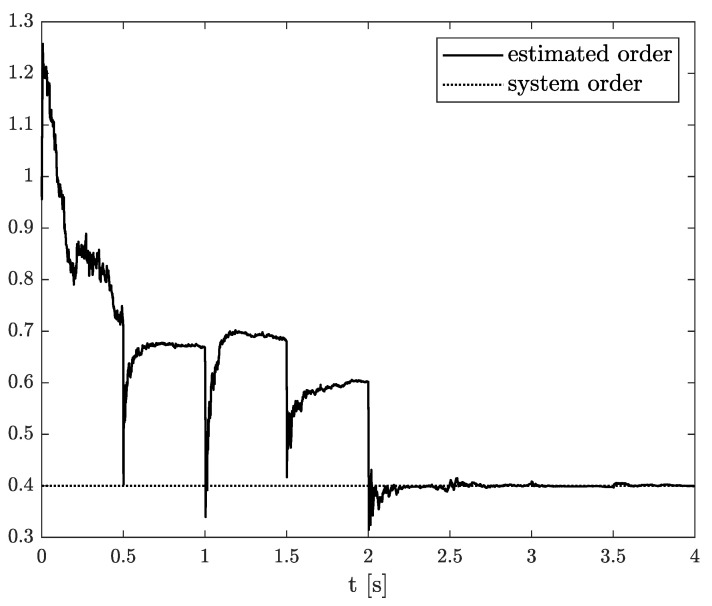
Original and estimated order from Example 1.

**Figure 7:**
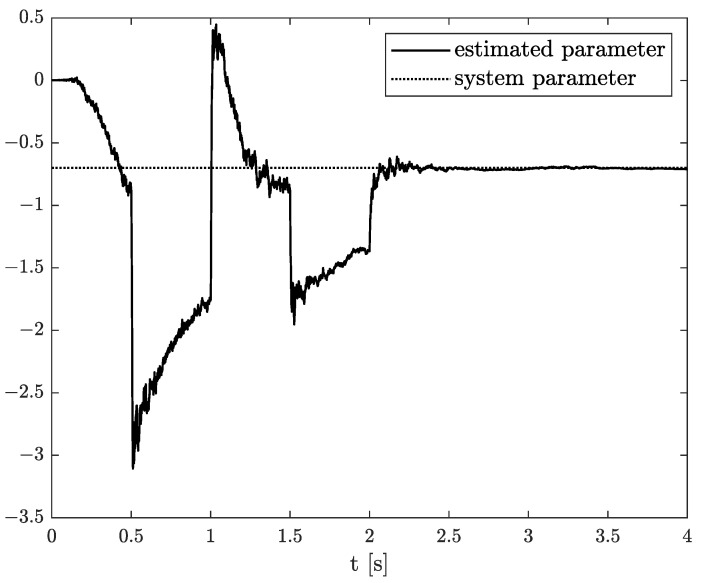
Original and estimated parameters from Example 1.

*The estimation problem is defined as the estimation of an unknown parameter in matrix A, defined as $w_{k}$, unknown system order $\alpha_{k}$, and state variable $x_{k}$, which is measured with some measurement noise. In this example, the results of state, order, and parameter estimation are presented in Figure 5, Figure 6 and Figure 7, respectively. As it can be seen in Figure 5, the state variable is accurately achieved as soon as the algorithm starts. However, looking at Figure 6 and Figure 7, it can be noticed that the order and parameter estimation accurately correspond to the simulation ones after 2 s.*

**Example** **2.** *This time, let us consider the linear Discrete Fractional Variable Order State-Space (DFVOSS) A-type system given by Equations (2), (3) and (4), where*

(61) $$A=-0.5,B=1,C=1,\alpha_{k}=0.6$$

**Figure 8:**
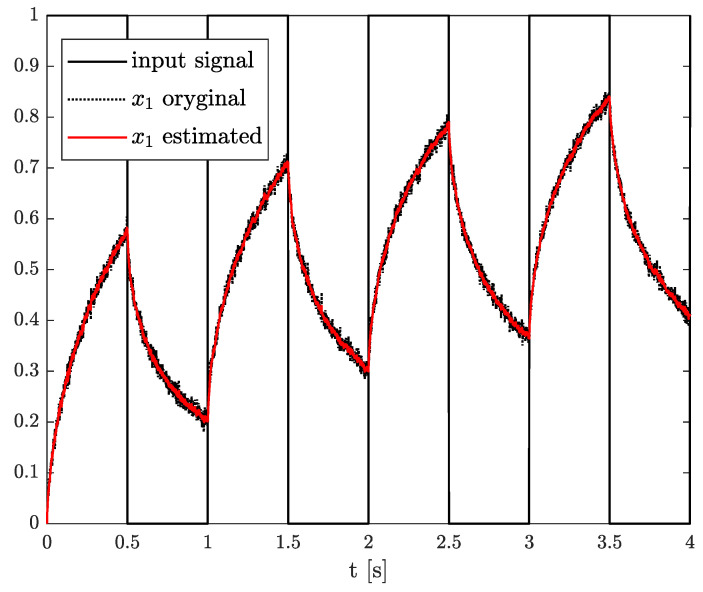
Original and estimated state variables from Example 2.

**Figure 9:**
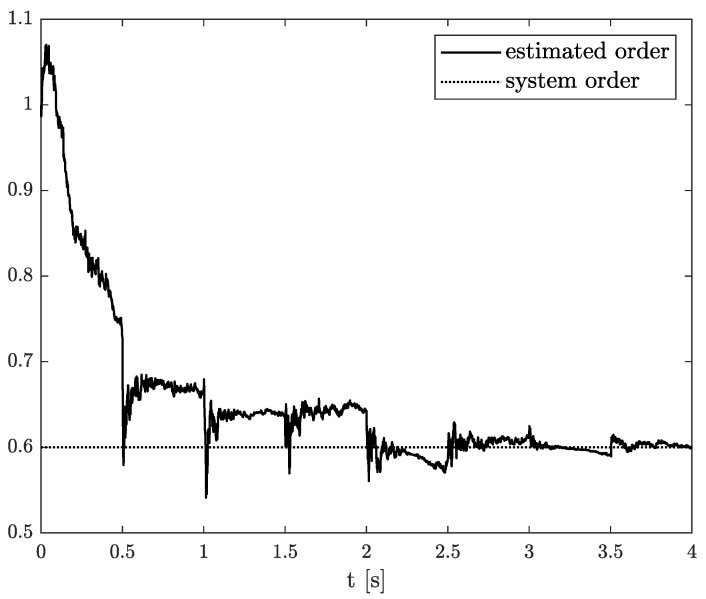
Original and estimated order from Example 2.

**Figure 10:**
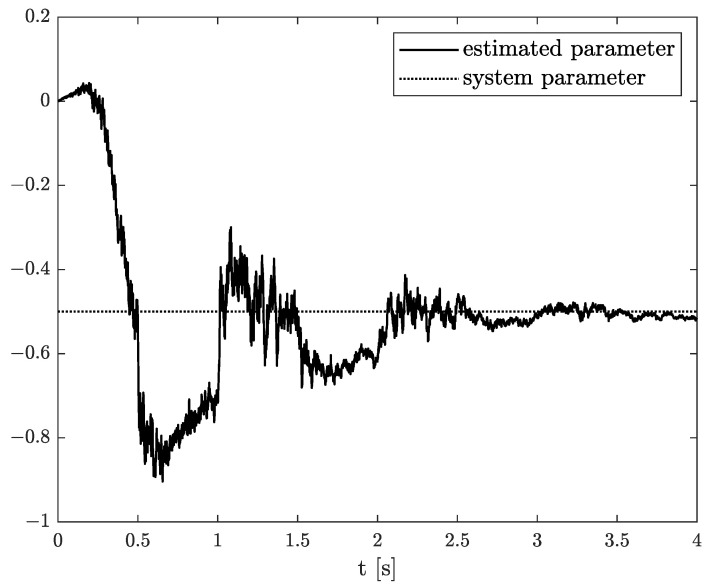
Original and estimated parameters from Example 2.

*The results of the state, order, and parameter estimation are presented in Figure 8, Figure 9 and Figure 10, respectively. Similarly to Example 2, the estimation of state variable corresponds to the system variable beginning with a simulation start point. Despite close values of order and parameter, their simulation values were achieved approximately at 2 s as well.*

**Example** **3.** *Now, let us take the next linear Discrete Fractional Variable Order State-Space (DFVOSS) A-type system given by Equations (2), (3) and (4), where*

(62) $$A=-0.8,B=1,C=1,\alpha_{k}=0.3$$

**Figure 11:**
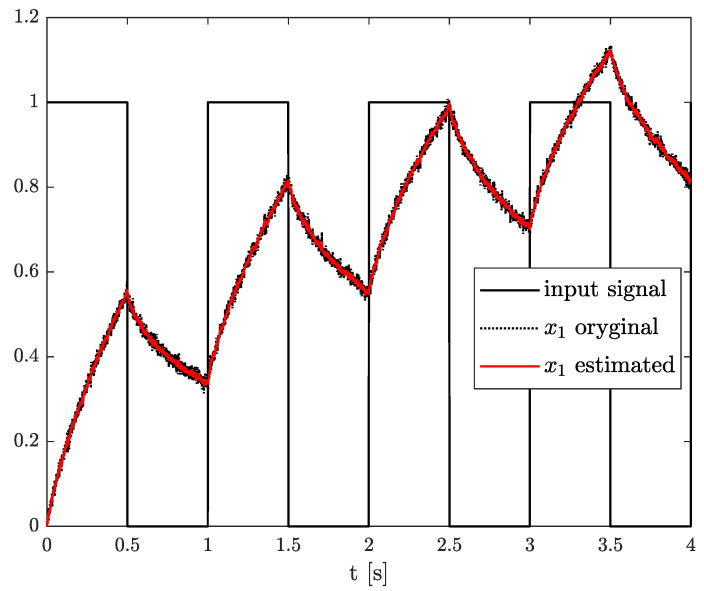
Original and estimated state variables from Example 3.

**Figure 12:**
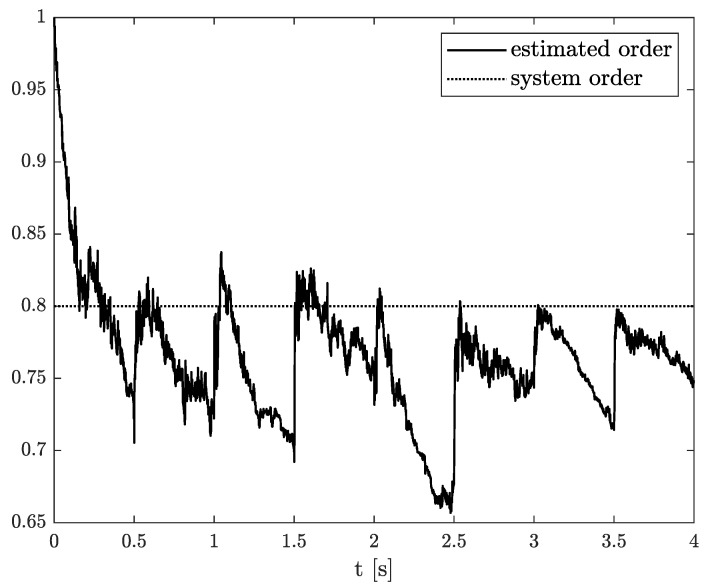
Original and estimated order from Example 3.

**Figure 13:**
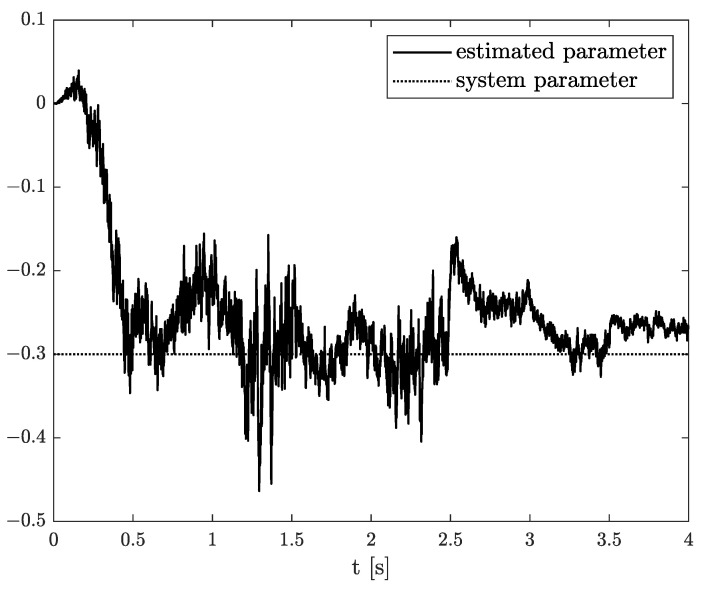
Original and estimated parameters from Example 3.

*In this case, the results of state, order, and parameter estimation are presented in Figure 11, Figure 12 and Figure 13, respectively. Analysing these results, it should be noticed that both order and system parameters were less than those in the previous examples. However, as was shown in Figure 13, the parameter and order estimations correspond to a constant system value with a small discrepancy but starting with 0.5 s.*

*It should be strongly highlighted that, in all distinguished examples, the proposed Triple Estimation algorithm was successfully used to determine the state, order, and parameter values of a simulated fractional order system with system and measurement noises.*

## 5. Conclusions

In this paper, the Triple Estimation algorithm was presented. Its main area of use is to simultaneously estimate the fractional systems’ states variables, parameters, and orders. Moreover, its accuracy was validated on sets of numerical examples under common, predefined algorithm configuration parameters. It should be mentioned that the results can strictly depend on parameters of estimators, especially parameters of Unscented Fractional Kalman Filter in the order estimation. The proposed Triple Estimation algorithm is composed of three Kalman Filter blocks with appropriate data interconnections between each other. The next huge advantage of the proposed algorithm comes out directly from its fractional variable-order behaviour, which means that it can be adapted to estimate the fractional variable-order systems with non-stationary parameters. This means that such an algorithm can be treated as a convenient tool for estimation, identification, and analysis of a wide range of fractional constant and variable order systems. For simplicity, in this paper, the estimation of a system with one state variable was considered. However, there are no restrictions on using the algorithm in more complicated systems with many state variables and many inputs and outputs. Obviously, the problem of the robustness of the algorithm parameters can be more noticeable, but it still gives more tuning possibilities than joint and dual estimation algorithms.

A further area of use for the proposed algorithm can be found in the analyses of fractional noises, derived from different sources. (e.g., inertial measurement units (IMU) or temperature sensors). Potentially, when the temperature and heat flux impact appear in the sensors’ measurements, the fractional order character can be pointed out and estimation algorithms such as Triple Estimation will be necessary.

## Data Availability

Not applicable.
